# Functional connectivity alterations in women with subjective cognitive decline

**DOI:** 10.1177/25424823251328340

**Published:** 2025-06-25

**Authors:** Ashleigh F Parker, Cassandra Szoeke, Heather Kwan, Alexandre Henri-Bhargava, Jodie R Gawryluk

**Affiliations:** 1 Department of Psychology, University of Victoria, Victoria, BC, Canada; 2 Institute on Aging and Lifelong Health, University of Victoria, Victoria, BC, Canada; 3 Healthy Ageing Program, Faculty of Medicine, Dentistry and Health Sciences, The University of Melbourne, Parkville, VIC, Australia; 4 Department of Medicine (Division of Neurology), University of British Columbia, Victoria, BC, Canada; 5 Department of Neurology, Vancouver Island Health Authority, Victoria, BC, Canada; 6 Division of Medical Sciences, University of Victoria, Victoria, BC, Canada

**Keywords:** Alzheimer's disease, default mode network, frontal parietal network, functional connectivity, subjective cognitive decline

## Abstract

**Background:** Alzheimer's disease (AD) is an incurable neurodegenerative disorder, which disproportionately affects women. Along the continuum of dementia, those who experience subjective cognitive decline (SCD) are thought to be the earliest group at risk for development of AD. **Objective:** The current study investigated differences in functional connectivity between healthy older women and older women with SCD in multiple resting-state functional connectivity networks. This study also examined whether additional differences existed between women with and without SCD, in various demographic variables, lifestyle factors, and medical comorbidities. **Methods:** 3T high-resolution resting-state functional MRI (fMRI) scans were retrieved for 25 healthy older women and 25 older women who self-report SCD from the Women's Healthy Ageing Program (WHAP). A seed-based approach was executed in FMRIB's Software Library (FSL) to examine significant differences in functional connectivity within the default mode network (DMN), frontoparietal network (FPN), and salience network (SN) between groups. Group comparisons were conducted between women with SCD and healthy women on various demographic variables, lifestyle factors, and medical comorbidities. **Results:** Findings revealed significant increases in functional connectivity in the DMN and FPN in older women with SCD compared to healthy older women. **Conclusions:** Women with self-reported SCD had increased functional connectivity, with no significant differences were detected between groups on comparisons of various demographic variables, lifestyle factors, and medical comorbidities.

## Introduction

Alzheimer's disease (AD) is a progressive neurodegenerative disorder that affects 43.1 million older adults worldwide.^1^ It is crucial that methods for earlier detection and intervention to be developed to better manage care for these individuals.^2^

One potential avenue for research on pre-clinical AD involves a focus on subjective cognitive decline (SCD). People with SCD self-report declines in their cognition despite normal performance on traditional neuropsychological assessment measures and may be earliest in the continuum of dementia.^3^ The SCD-Initiative has also codified criteria labeled as “SCD-plus” which describes additional criteria that may represent individuals at a greater risk for developing AD in the future.^3^ Deriving biomarkers that can identify individuals likely to progress along the continuum to mild cognitive impairment (MCI) and AD is an imperative focus of current research.

It has been suggested that those with SCD may be experiencing functional dysconnectivity and not yet displaying cell loss that can be seen at later points on the cognitive continuum (when objective cognitive abilities and ability to carry out IADLs are impacted). Among functional neuroimaging techniques, functional magnetic resonance imaging (fMRI) has demonstrated promise as a valuable research tool resting-state to examine functional connectivity and network alterations in the brains of individuals with MCI or AD compared to healthy controls.^4^ Currently, fewer studies have been conducted examining differences in functional connectivity between individuals with SCD and healthy controls, and more research is needed to better understand how changes in brain function may reflect a potential biomarker for future development of AD.^5^

Resting-state fMRI measures spontaneous fluctuations in blood oxygen level-dependent (BOLD) signals in different brain regions at the voxel level.^6^ Although several analysis approaches exist, seed-analyses have been commonly used to examine differences in functional connectivity patterns in the brain.

The default mode network (DMN) is the most studied resting-state network in the literature for detecting disruptions in functioning in relation to the development of AD and other dementias. The DMN is associated with different types of functioning such as performance on self-referential tasks, emotional processing through the integration of external sensory stimuli, and recollection of previously learned information.^7^

In addition to the DMN, other functional connectivity networks such as the frontoparietal network (FPN) and salience network (SN)^8,9^ have demonstrated differences between healthy older adults and those with MCI or AD. The FPN is implicated in attention, decision making, working memory, and cognitive control.^10,11^ The SN is involved in social functioning, communication, and self-awareness through detection and integration of salient cognitive, emotional, and sensory stimuli in one's environment.^12–14^ During cognitive tasks, the SN will engage the FPN and disengage the DMN in order to focus on the task at hand. In contrast, during rest or passive states, the SN tends to exhibit the opposite pattern, with disengagement from the FPN and re-engagement with the DMN.^15^

A review by Viviano and Damoiseaux^5^ highlight how alterations in functional connectivity between those with SCD and healthy controls may be a sensitive method to detect future development of AD or other dementias when other indicators of dementia risk are not present during this preclinical phase. Findings across studies have been mixed, where those with SCD have either no difference,^16,17^ increased functional connectivity,^18,19^ decreased functional connectivity,^20–22^ or both increased and decreased functional connectivity^23–25^ when compared with healthy controls. Although there seems to be conflicting results when it comes to functional connectivity in those with SCD compared to healthy controls, it is hypothesized that these results may be influenced by the amount of time individuals have been living with SCD.^5^ It has been suggested that those who show increased functional connectivity reflect individuals who are in an early stage of SCD whereas lower functional connectivity reflects those who have experienced SCD for a longer time period,^26^ although longitudinal studies are needed to verify this theory.

Consideration of risk factors for cognitive decline alongside neuroimaging results may provide additional understanding of individuals with SCD. Although advanced age is the largest risk factor for future development of dementia, several potentially modifiable risk factors for dementia have been outlined.^27^ These potentially modifiable risk factors include less education, hearing loss, traumatic brain injury, hypertension, excessive alcohol consumption (>21 units/week), obesity (body mass index (BMI) ≥ 30), smoking, depression, social isolation, physical inactivity, diabetes, and exposure to air pollution.^27^ With the elimination of these 12 risk factors, it is estimated that up to 40% of dementias may be delayed or prevented.^27^ A study by Omura et al.^28^ found that individuals with SCD were more likely to report a higher number of risk factors associated with dementia compared to those without SCD. In fact, studies outside of the neuroimaging literature have found demographic variables, lifestyle factors, and health related concerns differ between those with SCD and healthy controls. Recent studies have investigated patterns of multimorbidity in SCD samples to examine whether these variables are associated with difficulties related to SCD.^29^ A systematic review by Li et al.^30^ discuss a number of different predictors of further cognitive deterioration in those who report SCD.^30^ Some of these predictors include factors outlined in SCD-plus criteria, lower education, presence of *APOE* ε4, older age, depression, anxiety, and current smoking.^30^ For instance, studies have reported that current but not former smokers with SCD have a higher risk of developing AD in the future compared to those who have never smoked.^29,31^ SCD has also been associated with depression and lower quality of life scores and experiencing symptoms of depression has also been associated with increased risk of conversion from SCD to amnestic MCI or dementia.^31–33^ The prevalence of reporting SCD was highest in those with depression (28.5%) and hearing loss (24.7%).^28^

Mixed findings have been reported regarding BMI and risk of AD conversion in those with SCD. Higher BMI has been associated with both lower risk^31,34–38^ and higher risk^39–42^ of converting from SCD to MCI or AD. It is hypothesized that lower BMI may be attributed to metabolic changes that take place in latent or preclinical AD, however, this mechanism is not fully understood.^43^

Increased physical activity has long been recognized for its benefits on brain health.^44,45^ In those with SCD, physical activity has also been reported to potentially slow conversion to MCI or dementia.^46,47^ Additionally, engagement in leisure activities and mentally stimulating tasks have been reported as protective factors for delaying cognitive decline and amyloid-β deposition.^46^

Limited research has been conducted on differences in functional connectivity across multiple networks in those with SCD compared to healthy controls. Although these studies have included cognitive scores and neuroimaging data, there has been a lack of available information on these participants apart from sex, education, and age. This limited availability of participant characteristics impeded researchers’ ability to confidently determine whether differences in brain metrics between these two groups are solely attributed to the experience of SCD.

The current study examined resting-state functional connectivity across three different networks as well as demographic and lifestyle variables between women with SCD and healthy women. Specifically, we examined resting-state functional connectivity of the DMN, FPN, and SN between groups. Based on previous research, it was hypothesized that women with SCD would show differences in resting-state functional connectivity compared to healthy women. Given that studies of SCD often do not provide much information on their SCD cohort, we also examine demographic variables, lifestyle factors, and medical comorbidities consistent with those outlined by Livingston and colleagues.^27^ We hypothesized that there would be minimal differences between women with SCD and healthy women, as participants recruited for this study were community-dwelling women experiencing healthy aging.

## Methods

### Participants

Data were obtained from the Women's Healthy Ageing Project (WHAP). The WHAP, initially named the Melbourne Women's Midlife Health Project is an ongoing longitudinal population-based study that began in 1991. The WHAP is an Australian-based project initiated in the early 1990s to examine factors influencing women's health during and after menopause.^48,49^ All participants were Australian Caucasian women. Initially, over 1897 women aged 45–55 were included to participate in the baseline cross-sectional study. To be deemed eligible for the longitudinal study, participants needed to have determinable menopausal status, defined as having had menses in the previous three months, an intact uterus with at least one ovary, and not taking oral contraceptives or hormone therapy. Of the 1897 women who participated in the baseline study, 779 women were deemed eligible, and 438 consented to participate in the longitudinal study. The WHAP research spans various areas, including quality of life, mental and cognitive health, cardiovascular health, musculoskeletal health, lifestyle, women's health, and hormonal transitions. To date, this longitudinal study cohort has maintained nearly 50% retention over 30 years of follow-up assessments. Additional information regarding the recruitment and development of this research program have been previously published.^48,49^ Ethics approval for secondary data analyses were provided by the University of Victoria Human Research Ethics Board.

**Figure 1.**
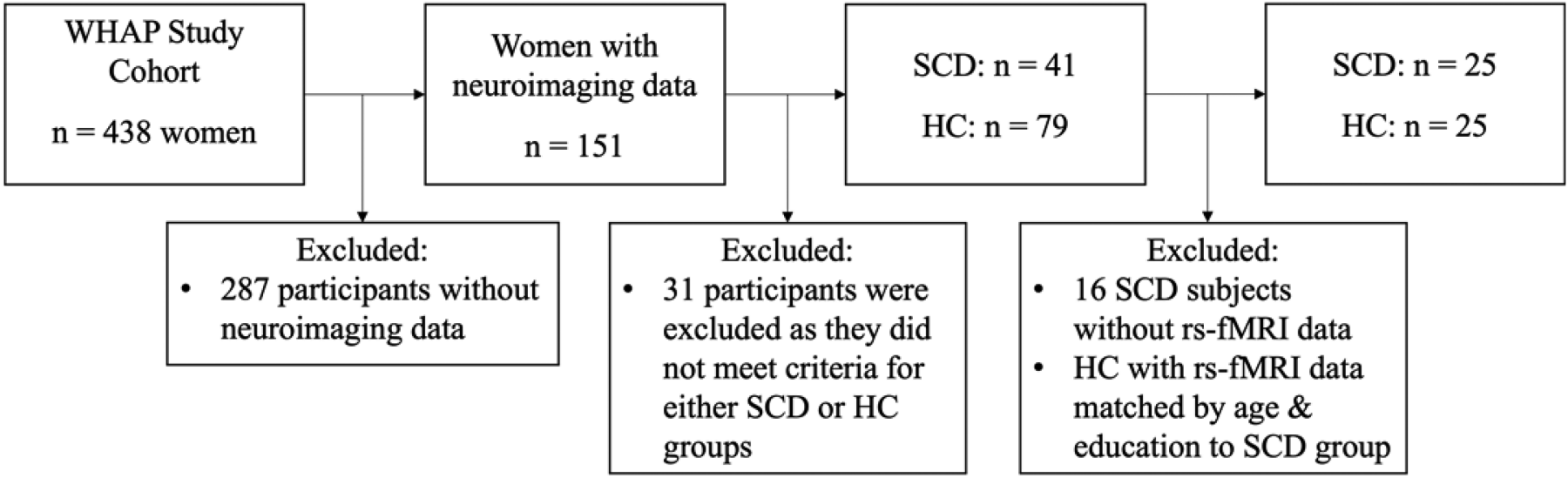
Flow diagram of participant selection for study 2. WHAP: Women's Healthy Ageing Project; SCD: subjective cognitive decline; HC: healthy controls.

The data utilized in the present study was collected between 2012–2015 and includes MRI data as well as neuropsychological, psychosocial, and lifestyle data. The participants from the WHAP were then sorted into one of two groups, SCD or healthy controls. A flow chart of participant selection is shown in Figure 1. Both the SCD and healthy control groups were closely modeled after those in the cohorts described in ADNI-2.^50^ All SCD participants self-reported a significant memory concern and achieved a score of 0 on the Clinical Dementia Rating (CDR). All control participants were free of memory complaints and deemed cognitively normal based on clinical assessments by the site physician showing an absence of significant impairment in cognitive functioning and performance of daily activities. Both the participants in the control and SCD groups exhibited normal memory function on the Logical Memory II subscale of the revised WMS (≥9 for 16 years of education and above, ≥ 5 for 8–15 years of education, and ≥3 for 0–7 years of education), a MMSE score between 24 and 30 (inclusive), and a CDR of 0.

### Image acquisition

MRI data were retrieved from the WHAP. All images were acquired on 3 Tesla Philips MRI scanners. Whole-brain anatomical MRI scans were acquired sagittally, with a T1-weighted MPRAGE sequence, with the following parameters: a repetition time (TR) of 7 ms, an echo time of 3 ms, voxel size of 1 × 1 × 1.2 mm, and a flip angle of 9°. fMRI scans were obtained during resting state (with eyes open). T2*-weighted, gradient EPI data were acquired continuously with an 8-channel birdcage radio frequency head coil. Each scan generated 180 volumes of whole-brain, 41-slice acquisition (TR = 3000 ms, TE = 30 ms, flip angle = 90°, voxel size = 3.0 mm^3^) for a 6 min duration acquisition. Participants were asked to keep eyes open and fixed on a projected cross hair.

### Data analyses

#### Image preprocessing

All MRI data obtained from the WHAP were in DICOM format. All structural images were converted from DICOM to NIFTI format using dcm2niix in the MRIcroGL application.^51^ All analysis steps were performed using tools within the Functional MRI of the Brain Software Library (FSL) version 6.0 (Analysis Group, FMRIB, Oxford, UK, http://fsl.fmrib.ox.ac.uk).^52^ Non-brain tissue in the raw T1 images was removed using the automated Brain Extraction Tool,^53^ followed by manual verification and optimization for each subject. The FEAT function was used to pre-process the data (including skull removal with BET and motion correction with MCFLIRT). No smoothing was applied. Registration of the functional data to the high-resolution structural image was carried out using the boundary-based registration algorithm.^54^ Next, registration of the high-resolution structural images to standard space was carried out using FLIRT^55,56^ and then further refined using FNIRT nonlinear registration.^57,58^

#### Seed-based resting state fMRI functional connectivity analysis

A seed-based approach was used to examine functional connectivity in the DMN, FPN, and SN. Three separate regions of interest (ROIs or seed-regions) were created in order examine each of these functional networks. These regions were modeled after previously used ROIs as documented in the literature, including the left posterior cingulate for the DMN,^59^ the right inferior parietal sulcus for the FPN,^60^ and the right dorsal anterior cingulate cortex for the SN.^61^ For each of these brain structures, a spherical ROI with a 5 voxel (5 mm) radius was generated, with the center point anchored to the corresponding MNI coordinates of each brain region as reported in the literature. Each of the created ROIs were generated using the MNI-152 T1 1 mm brain as the template.

Each of these created ROIs were then registered to individual space. Next, the FEAT function was used to examine each of the networks using the corresponding ROIs as well as regress out the lateral ventricle signal to correct for confounding noise.^17,23,62^

Specifically, the mean BOLD signal time series was extracted from each of the seed regions and used as the model response function in a general linear model. This allowed for examination of functional connectivity in the DMN, FPN, and SN respectively, through the detection of voxels with timeseries that correlated with that measured in each of the seed regions. The time-series statistical analysis was then carried out using FILM with local autocorrelation correction.^63^

Finally, a higher-level between-group analysis was conducted to compare resting state functional connectivity in each of the networks (DMN, FPN, and SN) between the SCD group and controls. The higher-level analysis was carried out using a fixed effects model, by forcing the random effects variance to zero in FLAME (FMRIB's Local Analysis of Mixed Effects).^64–66^ Z (Gaussianised T/F) statistic images were thresholded non-parametrically using clusters determined by Z > 3.0 and a (corrected) cluster significance threshold of *p *= 0.05.^67^ The regional anatomy showing significant alterations in functional connectivity were identified using the Harvard-Oxford Cortical Structural Atlas.

### Demographics analysis

#### Education

Education was further categorized into five distinct groups, those with 16 or more years of education, those with 13–15 years of education, those with 9–12 years of education, those with 7–8 years of education, and those with 6 or less years of education.

#### Smoking

Participants were asked whether they are currently smoking. Participants were categorized into two groups, those who currently smoke and those who do not.

#### Obesity (BMI ≥ 30)

The WHAP collected physical data to determine the BMI of their participants. This data was recorded numerically.

#### Alcohol use

Participants were asked to rate their alcohol use during follow up visits for the WHAP. Specifically, participants were asked “In the last 7 days, about how many alcoholic drinks have you consumed?”. The data from this question was categorized into seven categories, ≥ 15 drinks, 8–14 drinks, 7 drinks, 3–6 drinks, 2 drinks, 1 drink, or none.

#### Physical activity

Participants were asked to rate how often they engage in physical activity. Specifically, participants were asked “How often if at all do you participate in energetic physical activities which make you breathe harder, puff, or pant?”. Participants could choose between seven different options: everyday, 4–6 times per week, 2–3 times per week, once per week, a few times a month, less than once a month, or never.

#### Hypertension

Blood pressure data was collected from participants for both systolic blood pressure and diastolic blood pressure (both measured in mm Hg). Participants were classified as having hypertension if they self-reported taking anti-hypertensive medications or measured ≥140 mm Hg for systolic blood pressure or ≥90 mm Hg for diastolic blood pressure. Participants were categorized as being hypertensive or not based on the above criteria.

#### Diabetes

Participants were asked to self-report whether they have a diagnosis of diabetes. Data on fasting plasma glucose or HbA_1C_ was not collected.

#### Social isolation

Social isolation was measured through analyzing self-reported loneliness. Participants were asked “During the last week, how often have you been feeling lonely?”. This question is an item from the Affectometer 2.^68^ Participants were provided with five response options: most of the time, often, sometimes, hardly ever, or can’t say.

#### Traumatic brain injury

Participants were asked to self-report whether they have ever experienced a traumatic brain injury. Specifically, participants were asked “Have you suffered a serious head injury?”. If participants were to endorse a previous head injury, no data was collected on whether loss of consciousness was experienced.

## Results

**Figure 2.**
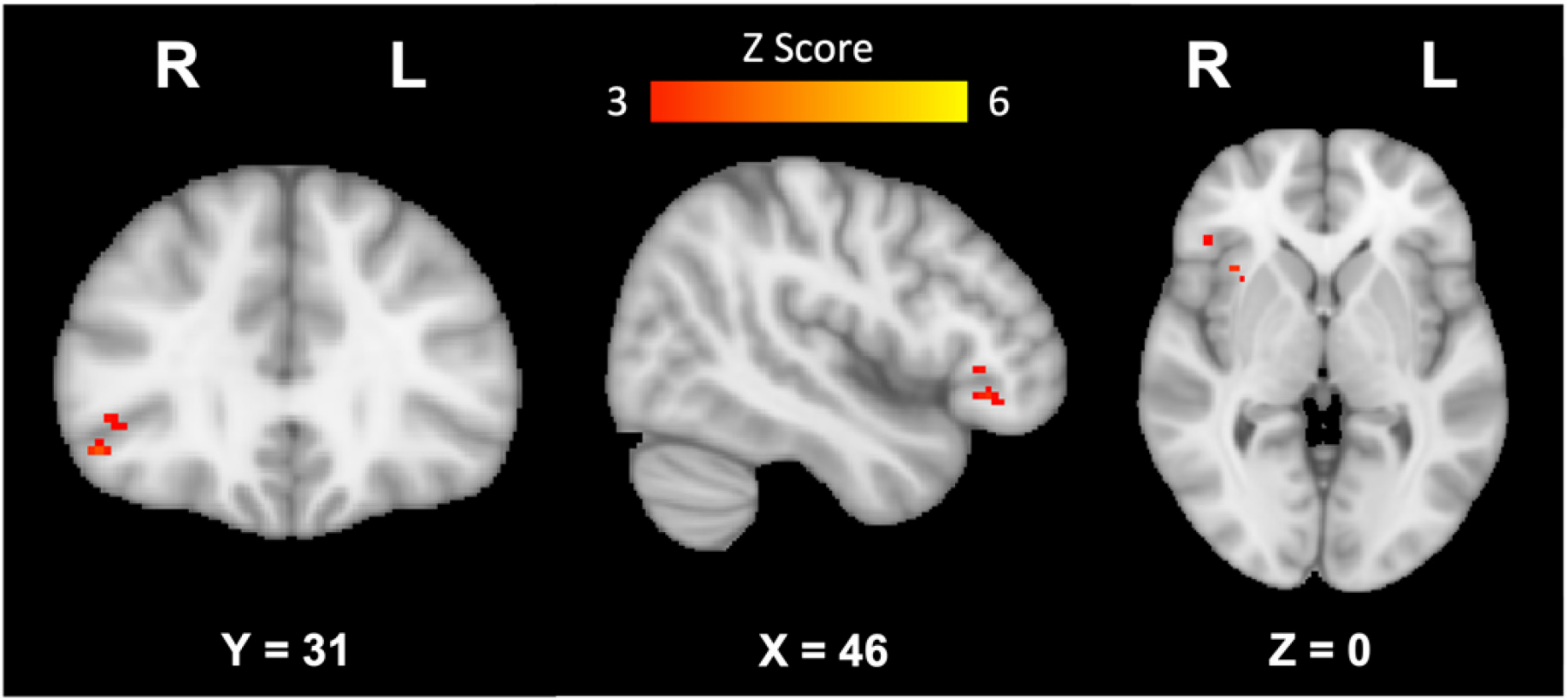
Brain regions showing increased functional connectivity in the DMN in participants with SCD compared to healthy controls. From left to right: sagittal, coronal, and axial slices of the brain displaying results of group level comparisons showing significantly increased functional connectivity in the DMN in women with SCD compared to healthy women; corrected for multiple comparisons, *p *< 0.05.

**Figure 3.**
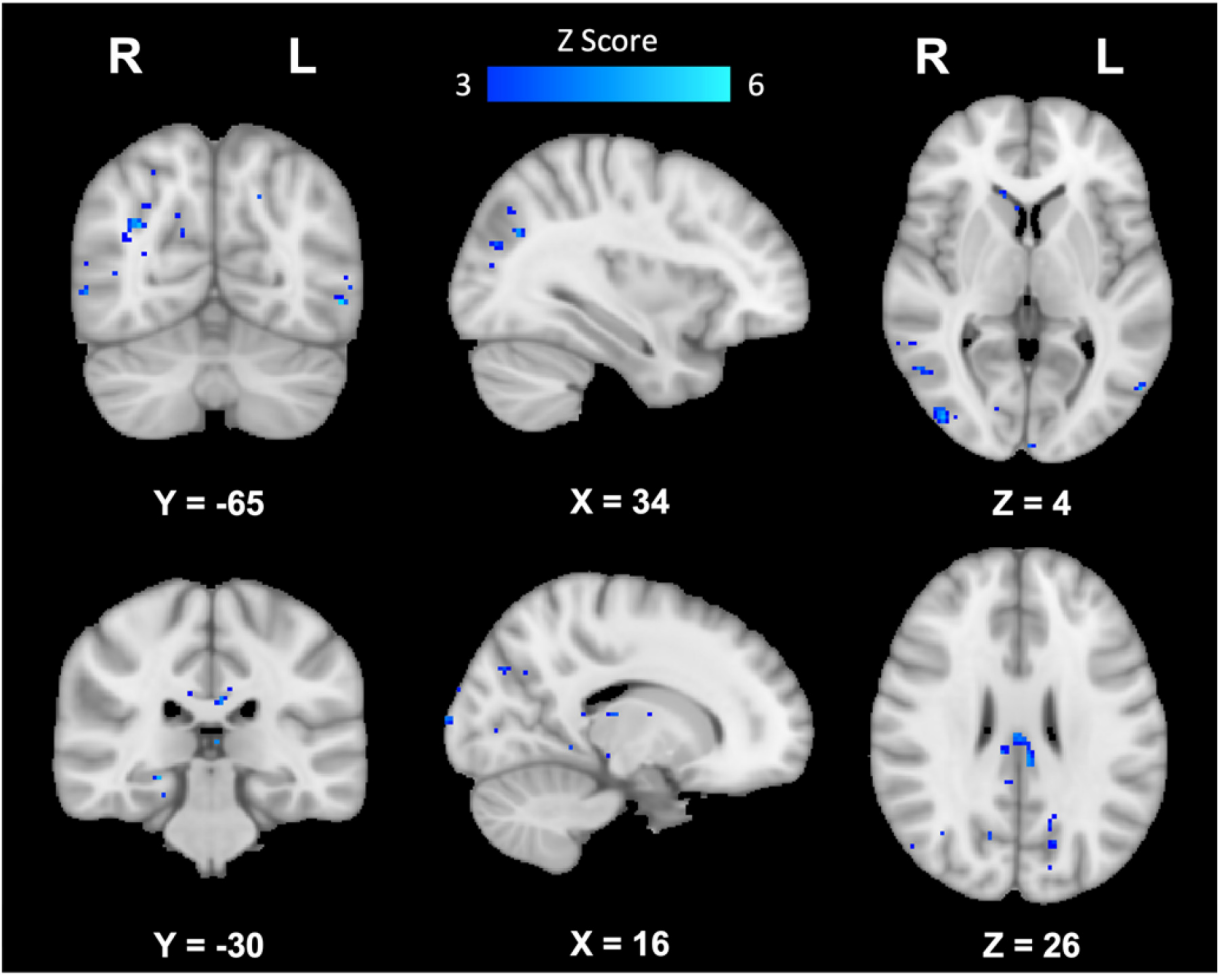
Brain regions showing increased functional connectivity in the FPN in participants with SCD compared to healthy controls. From left to right: sagittal, coronal, and axial slices of the brain displaying results of group level comparisons showing significantly increased functional connectivity in the FPN in women with SCD compared to healthy women; corrected for multiple comparisons, *p *< 0.05.

Resting state seed-based fMRI analyses revealed significant differences in functional connectivity between groups for the DMN and FPN where women with SCD showed increased functional connectivity in various regions of the brain compared to healthy women. In the DMN, women with SCD showed increased functional connectivity in the right frontal orbital cortex, frontal pole, inferior frontal gyrus (pars triangularis), and insular cortex (Figure 2, Table 1). In the FPN, women with SCD showed increased functional connectivity in the left amygdala, right frontal pole, lingual gyrus, lateral occipital cortex (superior), parahippocampal gyrus, precuneus cortex, and bilateral cingulate gyrus (posterior), lateral occipital gyrus (inferior), and thalamus (Figure 3, Table 2). No differences in functional connectivity in the SN were found between women with SCD compared to healthy women.

**Table 1.** Brain regions showing increased functional connectivity in the DMN in participants with SCD compared to healthy controls.

		MNI Coordinates			
Brain Region	Laterality	X	Y	Z	Z score
Inferior Frontal Gyrus, pars triangularis	R	46	29	0	3.25
Frontal Pole	R	46	35	−10	3.11
Insular Cortex	R	36	18	0	3.59
Frontal Orbital Cortex	R	48	29	−8	3.99

Brain regions showing increased functional connectivity in the DMN in participants with SCD compared to healthy controls (min Z > 3.0; cluster significance: p < 0.05, corrected for multiple comparisons). Coordinates in the MNI-152 standard space image are given.

**Table 2.** Brain regions showing increased functional connectivity in the FPN in participants with SCD compared to healthy controls.

		MNI Coordinates			
Brain Region	Laterality	X	Y	Z	Z score
Parahippocampal Gyrus	R	22	−35	−16	3.30
Cingulate Gyrus (Posterior)	L	−4	−36	23	4.38
Cingulate Gyrus (Posterior)	R	2	−23	25	3.54
Thalamus	R	18	−23	12	3.08
Thalamus	L	−4	−18	13	4.04
Lateral Occipital Cortex (Superior)	R	35	−65	28	3.79
Lateral Occipital Cortex (Inferior)	R	42	−84	4	3.96
Lateral Occipital Cortex (Inferior)	L	−54	−65	−5	3.54
Lingual Gyrus	R	12	−44	−4	3.15
Frontal Pole	R	28	48	16	3.96
Amygdala	L	−22	−8	−10	3.21
Precuneus Cortex	R	19	−68	33	3.14
Insular Cortex	L	−38	−11	9	3.24
Caudate	L	−8	0	9	3.08
Hippocampus	R	25	−30	−9	4.01

Brain regions showing increased functional connectivity in the FPN in participants with SCD compared to healthy controls (min Z > 3.0; cluster significance: p < 0.05, corrected for multiple comparisons). Coordinates in the MNI-152 standard space image are given.

### Demographics

No significant differences were found between women with SCD and healthy women among variables of obesity, diabetes, hypertension, physical activity, social isolation, alcohol use, previous brain injury, and current smoking (Table 3).

**Table 3.** Participant demographics.

	HC	SCD	HC versus SCD
Age	69.80 ± 2.55	69.48 ± 2.87	*p *= 0.68^a^
Years of Education	14.24 ± 3.22	13.28 ± 3.61	*p *= 0.33^a^
Sex (M/F)	0/25	0/25	
Number of Pregnancies	3.52 ± 1.53	2.76 ± 1.36	*p *= 0.07^a^
Age at Final Menstrual Period	52.83 ± 2.32^b^	53.54 ± 1.40^c^	*p *= 0.44^a^
HRT Use Ever Count	0	1	
APOE e4	7^d^	1^e^	
BMI	26.42 ± 3.59	27.35 ± 5.23	*p *= 0.46^a^
Diabetic Count	1	1	
Hypertension Count	18	17	
Current Smoker Count	2	1	
TBI Count	2	2	
Alcohol Use		^f^	
≥15 drinks/week	1	3	*p *= 0.61^g^
8–14 drinks/week	0	2	
7 drinks/week	6	6	
3–6 drinks/week	5	2	
2 drinks/week	3	3	
1 drink/week	1	1	
None	9	7	
Physical Activity			
Everyday	1	0	*p *= 0.16^g^
4–6/week	2	0	
2–3/week	0	2	
1/week	5	7	
Few times/month	11	6	
<1/month	3	8	
Never	3	2	
Social Isolation			
Most of the time	0	0	*p *= 0.55^g^
Often	0	0	
Sometimes	2	1	
Hardly Ever	23	24	
Can’t Say	0	0	

^a^ p-value of t-test; ^b^n = 14; ^c^ n = 8; ^d^ n = 23; ^e^ n = 22; ^f^ n = 24; ^g^ p-value of chi-square test.

## Discussion

The current study represents an important step in characterizing functional connectivity and sociodemographic differences between older women who report SCD compared to healthy older women. The first aim of the present study was to determine whether there are functional connectivity differences between women with SCD and healthy women. Based on our previous work and review of literature, we hypothesized that functional connectivity would differ between these groups in all three of the networks investigated, including the DMN, FPN, and SN. Upon completion of the analyses, we found that older women who reported SCD showed increased functional connectivity in the DMN and FPN, while no decreases in functional connectivity were observed between these groups within these networks. Finally, our findings indicated no significant difference in functional connectivity between these groups in the SN. The second aim of this study was to examine whether these two groups differed on demographic variables, lifestyle factors, and medical comorbidities outlined by Livingston and colleagues^27^ as many studies of SCD do not provide detailed information on these variables in their study cohorts. As our participants were drawn from a healthy aging study cohort, we hypothesized that there would be minimal differences between women with SCD and healthy women, which was in line with our findings.

Our results are consistent with several studies that found increased functional connectivity in the DMN in individuals with SCD compared to healthy controls where those with SCD.^18,19,69–71^ While we did not find significant differences between those with SCD and healthy controls in the SN, a previous study has found increased functional connectivity in this network in those with SCD compared to healthy controls.^72^ We were challenged to find any previous study that examined functional connectivity differences in the FPN using resting-state fMRI in individuals with SCD. However, studies have found functional connectivity alterations between individuals with SCD and healthy controls in the FPN while using task-based fMRI. A review by Viviano and Damoiseaux,^5^ hypothesized that alterations in the FPN or SN may occur before differences are seen in the DMN. Our current results contrast this hypothesis as we saw increased functional connectivity in the DMN and FPN, while no differences were seen in the SN between groups. However, our results were based on resting-state fMRI protocols where as previous studies have seen alterations in the SN between those with SCD compared to healthy controls on task-based fMRI analyses.^73^

Broadly, in terms of cognitive theories of aging, the current findings align with the Scaffolding Theory of Aging and Cognition – Revised (STAC-R) which describes functional compensation mechanisms (i.e., increased functional connectivity) as a means of over-recruiting additional cortical areas to support neural circuitry that is underperforming.^74^ Our findings of increased functional connectivity in frontal regions, specifically within the DMN also corresponds with the Posterior-Anterior Shift in Aging (PASA) model. The PASA model describes a shift in functional activity from the posterior to frontal regions of the brain.^75^ However, the study of these cognitive theories are mainly done in groups comparing older adults to younger adults. Further research is necessary to better understand the relevance of these cognitive theories when evaluating brain-based differences between older adults who report SCD and those without cognitive complaints.

Previous studies comparing those with SCD to healthy controls have primarily focused on age and education as differentiating factors, without thoroughly examining other demographic or lifestyle variables. In the current study, we sought to investigate whether additional differences existed between these two groups other than the experience of SCD by including comparisons on potentially modifiable risk factors outlined by Livingston and colleagues.^27^ We specifically examined variables including obesity, diabetes, hypertension, physical activity, social isolation, alcohol use, previous brain injury, and current smoking. In line with our hypothesis, we did not observe any significant differences between women with SCD and healthy women across these variables. This finding is intriguing since we observed differences in functional connectivity between these two groups based solely on the self-reporting of SCD, while no other significant differences were identified on these demographic variables, lifestyle factors, and medical comorbidities.

This current study possesses several notable strengths. A fundamental strength of this study was the comprehensive examination of resting-state functional connectivity differences across three distinct networks—the DMN, FPN, and SN—between women with SCD in comparison to healthy women across. To our knowledge, this is the first study to examine differences in multiple networks between individuals with SCD and healthy controls in a sample comprising of women. By specifically examining functional connectivity differences in older women with SCD, the findings of this study enhance the current understanding of cognitive health in women. Using resting-state fMRI was another strength of this study and can ensure inclusivity in data collection. This approach is well-suited for the study of individuals with compromised cognitive abilities. While task-based fMRI remains a valuable tool for investigating alterations in functional connectivity, it is important to acknowledge that individuals with advanced dementia often encounter challenges when attempting to complete complex tasks, rendering the resting-state approach as a more viable and informative alternative. Another strength of this study was the incorporation of demographic variables, lifestyle factors, and medical comorbidities. The inclusion of these variables allowed for a comprehensive characterization of the sample and confirmed that among these variables considered, the only discernible distinction between these two groups was the self-reported experience of SCD.

There were also several limitations of this current study that may help inform the direction of future research in this area. First, there was a limited statistical power due to the sample size of the study, however, we included the maximum number of participants available in the WHAP as participants were required to have undergone both structural and functional MRI. However, the small sample size may limit the conclusions that can be drawn from the current results and future studies should replicate these results with a greater number of participants. Second, this study employed a cross-sectional design. Future research would benefit from using longitudinal designs that could elucidate the functional connectivity changes between these groups over time. Third, the current study only included female-bodied healthy women and female-bodied women with SCD. Future studies would benefit from including a broader range of groups across the cognitive continuum, such as healthy controls, male-bodied individuals, and individuals with varying levels of cognitive decline such as SCD, MCI, and AD. By incorporating each of these groups, researchers may gain a more comprehensive understanding of the differences in functional connectivity across the cognitive continuum. Future studies could also examine correlations between functional connectivity and cognitive performance to better understand these relationships. A fourth limitation was the absence of specific information regarding participants’ experience of SCD. Despite participants completing a multitude of cognitive tests and lifestyle questionnaires, this dataset lacked detailed information outlined by the SCD-I. Future research should consider incorporating specific information to gain a better understanding of the relationship between SCD and functional connectivity alterations. Recent research has suggests that the experience of persistent SCD over several years should be included in the SCD-plus criteria.^76^ Liew^76^ conducted a study investigating differing trajectories of SCD and their impact on conversion rates to MCI or dementia. The findings revealed that older individuals who reported persistent SCD had a probability greater than 75% of developing MCI or dementia in 10 years.^76^

### Conclusion

This study aimed to investigate the differences in functional connectivity networks, specifically the DMN, FPN, and SN, between healthy older women and older women with SCD. The results revealed increased functional connectivity in older women with SCD in the DMN and FPN compared to healthy older women. Notably, no decreases in functional connectivity were found between the two groups across networks, and no significant differences in functional connectivity were detected in the SN. The observed findings of increased functional connectivity (rather than decreased functional connectivity) between groups may be attributed to characteristics of the participant sample which comprised of community-dwelling women who are likely in the earlier stages of experiencing SCD. Further, no differences emerged between groups in various demographic variables, lifestyle factors, and medical comorbidities. The absence of differences between groups on these variables highlights how the only distinction between the two groups was the self-report of SCD. Future studies should also incorporate demographic variables, lifestyle factors, and medical comorbidities, as well as capture specific information regarding SCD-plus criteria as well as the duration of time that individuals have been experiencing SCD. The inclusion of this information would contribute to a more fulsome description of the individuals included in the SCD group and provide further insights into the relationship between functional connectivity and the experience of SCD. Overall, the findings of this study provide valuable insights into the neural mechanisms underlying SCD and highlights the importance of considering functional connectivity alterations in understanding preclinical manifestations of AD.
